# Assessment of RF Electromagnetic Exposure to Car Driver from Monopole Array Antennas in V2V Communications Considering Thermal Characteristics

**DOI:** 10.3390/s25103247

**Published:** 2025-05-21

**Authors:** Shirun Wang, Mai Lu

**Affiliations:** Key Laboratory of Opto-Electronic Technology and Intelligent Control of Ministry of Education, Lanzhou Jiao Tong University, 88 West Anning Road, Anning District, Lanzhou 730070, China; 12232066@stu.lzjtu.edu.cn

**Keywords:** V2V communications, monopole array antenna, electromagnetic exposure, COMSOL Multiphysics, temperature rise

## Abstract

Vehicles are rapidly evolving into objects of intelligent interconnection. Vehicle-to-Vehicle (V2V) communications enable the interconnection between vehicles, while also leading to new electromagnetic exposure scenarios. This paper integrates a monopole array antenna into a shark-fin antenna on the car roof for V2V communications and evaluates the specific absorption rate (SAR) and temperature rise of a human body in a smart mobility communication scenario operating at 5.9 GHz. The V2V antenna is modeled and placed on a 3D vehicle model using COMSOL Multiphysics (v.6.2) to numerically estimate the SAR in the head and body regions of the human body model (adult male) inside the vehicle. Both the localized and whole-body 30 min average SAR are lower than the International Commission on Non-Ionizing Radiation Protection (ICNIRP) occupational restrictions for electromagnetic field exposure from 100 kHz to 6 GHz, being equal in the worst-case scenario to 0.981 W/kg (for the head), which is 9.81% of the ICNIRP limit (10 W/kg), and 0.008728 W/kg (for the whole-body average), which is 2.18% of the ICNIRP limit (0.4 W/kg). The 30 min average human core temperature rise is 0.055 °C, which is 5.5% of the ICNIRP limit. This indicates that, in typical automotive scenarios, the electromagnetic exposure from a monopole array antenna for V2V communications does not pose threat to the human body. This study provides knowledge related to emerging exposure scenarios in intelligent mobility communication, which is beneficial for evaluating possible health impacts and designing public health management policies.

## 1. Introduction

With the advent of autonomous driving and connected driving, smart vehicles are rapidly changing the way we move, bringing new innovative services and applications to improve safety and enable more efficient traffic management [1].

**Figure 1:**
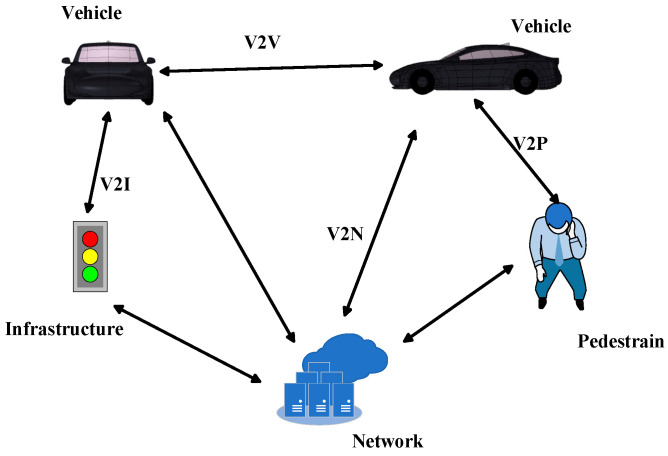
V2X communication scenario.

Such applications will be made possible by Vehicle-to-Everything (V2X) communications, which incorporates Vehicle-to-Infrastructure (V2I), Vehicle-to-Vehicle (V2V), Vehicle-to-Pedestrian (V2P), and Vehicle-to-Network (V2N) communications [2]. V2I Communications enable real-time data exchange with Roadside Units (RSUs) to optimize traffic signal phase timing and issue congestion alerts. V2V communications utilize 5.9 GHz Dedicated Short-Range Communications (DSRC) or Cellular Vehicle-to-Everything (C-V2X) protocols to enable millisecond-level emergency braking warnings and platooning functionalities. V2P communications ensure the safety of vulnerable road users through LTE/5G-enabled smart devices. V2N communications deliver cloud-based services via cellular networks, including real-time high-definition map updates, and fleet management system integration. V2X antennas are key components for vehicles to connect wireless networks, remote diagnosis, and safety warnings. Figure 1 shows the V2X communication scenario [3].

As the radio frequency (RF) antennas of vehicles become more intelligent and diversified, the in-vehicle electromagnetic environment has become increasingly complex. According to statistical data, the average daily driving duration for a member of the general public on weekdays is roughly about 1–2 h [4]. In the complex RF electromagnetic environment of vehicles, drivers and passengers may be exposed to a large number of RF electromagnetic fields, potentially posing health and safety risks. In particular, drivers endure prolonged RF exposure.

In 2011, Harris et al. calculated the specific absorption rate (SAR) values of adults and children exposed to wireless communication systems (including UMTS, WiMax, and Bluetooth) in the shielded environment inside a vehicle, and all the values were lower than the basic limits in the guidelines of the International Commission on Non-Ionizing Radiation Protection (ICNIRP) [5]. In 2011, Wessapan et al. conducted a numerical analysis on electromagnetic exposure using a realistic human model. Their study evaluated how different operating frequencies (900 MHz and 1800 MHz) and antenna-to-head distances influenced temperature field distributions and specific absorption rate (SAR) distributions [6]. In 2020, Tognola et al. evaluated the dose of radio frequency electromagnetic fields (RF-EMF) absorbed by adult drivers inside vehicles equipped with a V2V monopole antenna operating in the ITS-5.9 GHz frequency band [7]. In 2022, Bonato et al. evaluated the exposure levels of adult human body models located at different positions around the vehicle on the road near the vehicle [8]. In both studies [7,8], the radiation dose absorbed by the human body consistently remained below the basic restriction limits recommended by ICNIRP for the 100 kHz–6 GHz frequency range. Specifically, the whole-body average specific absorption rate (SAR) did not exceed 0.08 W/kg, while the localized SAR in 10 g of tissue in the head and torso remained below 2 W/kg, and in the limbs, below 4 W/kg. In addition, Schilling et al. assessed the electromagnetic exposure from various radio devices inside vehicles at nine measurement locations based on the ICNIRP guidelines. The results showed that the maximum radiation levels of the ITS-G5 (windshield antenna), ITS-G5 (roof antenna), Bluetooth (external hands-free system), Wi-Fi (original integrated system), and Bluetooth (original integrated system) were all below the reference levels in the ICNIRP guidelines [9]. In 2024, Shang et al. evaluated human electromagnetic exposure from the GPS antennas mounted on electric vehicles. The calculated exposure levels of the research remained well within the safety thresholds established by the ICNIRP, suggesting minimal health risk [10]. In 2024, Benini et al. evaluated the whole-body specific absorption rate from 5.9 GHz V2V and V2I communications in urban settings using human models representing children and adults. The maximum SAR was 4.9 × 10⁻^4^ W/kg, occurring within 10 m of multiple transmitting vehicles, with adults experiencing higher exposure than children [11]. In 2024, Yang et al. evaluated human RF exposure from 5.9 GHz V2X systems (including RSUs, OBUs, and Tesla Model S technologies) using numerical simulations and experimental measurements, confirming all exposure levels remain significantly below the ICNIRP limits [12]. In 2025, Stroobandt et al. [13] investigated auto-induced uplink (a-UL) RF-EMF exposure in 4G and 5G networks across 282 microenvironments in seven European countries, using a novel methodology with the application QualiPoc. Their results showed that 5G transmit powers were 3.3 dB lower than 4G, with the lowest levels in TDD frequency bands due to low uplink duty cycles, and base station density was a key predictor of exposure.

There are two main wireless access technologies for V2X communications: IEEE 802.11p (or its European version ITS-G5) and Cellular Vehicle-to-Everything (C-V2X), both working at 5.9 GHz [14]. C-V2X was standardized by 3GPP Release 14 in 2017, replacing Dedicated Short-Range Communication (DSRC), using LTE as the underlying technology to connect vehicles to everything [15]. This study primarily focuses on C-V2X, which has introduced new electromagnetic exposure scenarios. As with V2V communications, investigating human electromagnetic exposure in this specific communication context and conducting safety assessments can help mitigate potential risks to the personal safety of vehicle users. Currently, safety assessments regarding the exposure of vehicle drivers to RF electromagnetic environments are still insufficient, especially concerning the impact of in-vehicle RF V2V antennas on the human body. Previous research on V2V communications has also only concentrated on monopole antennas or patch antennas [7,8,16], with limited research on the thermal effects on the human body when exposed to a 5.9 GHz radiation source for 30 min. In this paper, an electromagnetic-thermal coupling computational model is established to evaluate human RF electromagnetic exposure to a monopole phased array antenna that meet V2V communication requirements has been carried out, using COMSOL Multiphysics to conduct a simulation based on the finite element method. Compared to other simulation calculation methods, this approach can better accommodate complex geometries, improve accuracy, and offer greater flexibility when dealing with complex boundary conditions. We simulate the E-field and SAR distributions in a human body model caused by electromagnetic exposure, the whole-body E-field and human core temperature distributions after 30 min of electromagnetic irradiation. These results are subsequently compared to the latest international standard, the ICNIRP (2020) [17].

The remainder of this paper is organized as follows. Section 2 presents the computational models employed in this study, including the human body model, vehicle model, and antenna model. Section 3 elaborates on the theoretical foundations of our research. Section 4 discusses the results with reference to the ICNIRP guidelines. Finally, Section 5 provides conclusions and proposes potential directions for future research.

## 2. Model Building

### 2.1. V2V Antenna Model

V2V antennas are crucial for implementing intelligent transportation systems and are among the key components of smart vehicles. They are typically omnidirectional, able to transmit and receive signals in all directions. Since vehicles may communicate with objects at a long distance, such as infrastructure or base stations several kilometers ahead, the V2V antenna needs to have high gain and good directional control capabilities. It should be able to flexibly adjust the beam direction according to communication requirements, reducing interference in other directions and improving communication efficiency and quality. A phased array antenna is one where the direction of the main lobe (the direction of maximum radiation) or the shape of the radiation pattern is primarily determined by the relative phase of the currents in the individual elements, which allows the main lobe direction to adjust continuously according to communication needs. By leveraging the principle of coherent superposition, phased array antennas can achieve higher gains [18]. This is highly advantageous for V2V communications that require long-range transmission.

**Figure 2:**
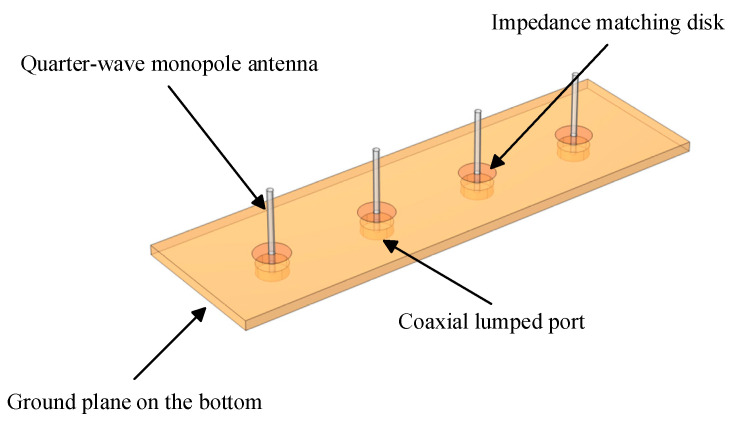
The structure of the V2V antenna.

To ensure both omnidirectionality and high gain, an array consisting of four quarter-wavelength monopole antennas is constructed in this study, which can adjust the beam direction flexibly according to communication needs. The structure is shown in Figure 2, meeting V2V communication requirements. Based on a center frequency of 5.9 GHz for V2V communications and Equation (1), the arm length of each monopole antenna can be calculated and optimized to 12.7 mm, with a radius of 0.65 mm.

(1) $$\lambda =\frac{c}{f}$$

The antenna arms are mounted on a dielectric substrate of which the dimensions are 30 mm × 100 mm × 2 mm, with the bottom side serving as the ground plane. The ground plane should have a diameter or side length of at least λ/2 to ensure proper radiation characteristics. The relative permittivity (ε) of the dielectric substrate is 3.38. Each antenna is fed by a coaxial lumped port with a characteristic impedance of 50 Ω. We assessed the exposure dose induced by V2V antenna operating at an emitted power level of 33 dBm, the maximum permissible level under European Union regulations [19].

The spacing between the antenna elements is 0.47 times the free-space wavelength, which results in relatively high gain and low sidelobe levels, while also preventing the generation of unwanted grating lobes. Metal circles are fabricated on the top of the substrate and connected to each monopole radiator element. These circular patches are employed to compensate for the inductance of the monopole antenna and achieve impedance matching with the reference impedance of 50 Ω. The parameters of the antenna material are shown in Table 1.

The antenna arms are made from copper, due to its excellent conductivity. The antenna employs coaxial feed. To ensure the coaxial line impedance is 50 ohms, suitable for RF communication systems, the relative permittivity of the coaxial lumped port is set to 3.38.

**Figure 3:**
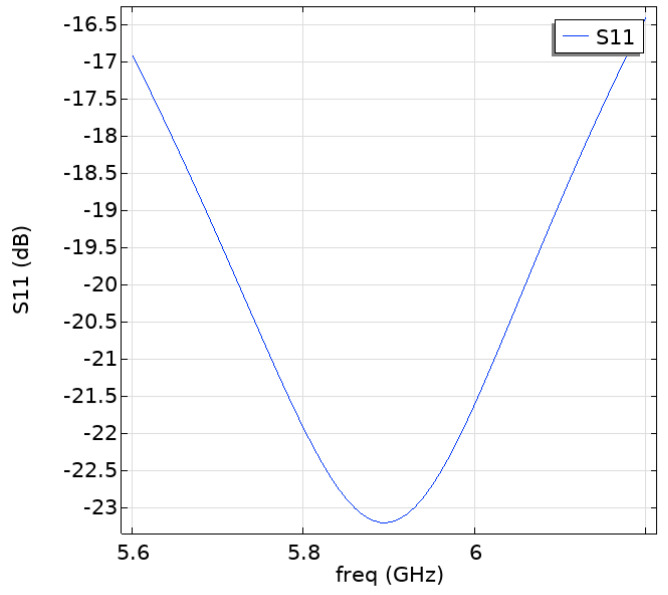
Antenna S11 parameter.

Figure 3 shows the S11 parameter of the antenna with an input power of 33 dBm. The parameter S11 is reflection coefficient, representing the ratio of incident electromagnetic waves that are reflected. It can be observed that the resonance frequency of the antenna is located at 5.9 GHz, where the return loss is below −23 dB. Lower return loss ensures good signal transmission and reception. Therefore, the antenna exhibits good return loss.

All elements are excited with the same element phase α, using the same magnitude and arithmetic phase variation (0, α, 2α, 3α) during the simulation, as shown in Table 2. Specifically, the excitation phase of lumped port 1 is fixed at 0° as the reference phase, while the excitation phases of lumped ports 2, 3, and 4 are sequentially incremented by α. By adjusting the phase differences among these ports, the radiation pattern of the antenna array can be controlled, thereby enabling beam steering.

When all antenna elements are simultaneously excited, the radiation pattern exhibits enhanced directivity, which can effectively achieve omnidirectional signal coverage. This is crucial for the communication effectiveness of smart vehicles. When the phase difference between adjacent antenna elements is fixed, a gradient distribution is introduced, adjusting the interference conditions of the waves, enhancing coherence in specific directions while weakening coherence in other directions, forming a main beam in a specific direction in the far field. Different gradient distributions produce different main beam directions. When the unit phase is 0, all ports are excited with the same current phase, and the main beam simultaneously points in both the boresight (frontal) and end-fire (rear) directions, aligned with the normal direction of the antenna array, resulting in a more symmetrical radiation pattern. When the positive or negative of the unit phase changes (in the case where unit phase is ±90°, as shown in Table 2), the amplitude distribution of the wavefront remains unchanged, but the interference directivity of the wavefront changes, causing the main beam direction in the far field to undergo a symmetry flip.

**Figure 4:**
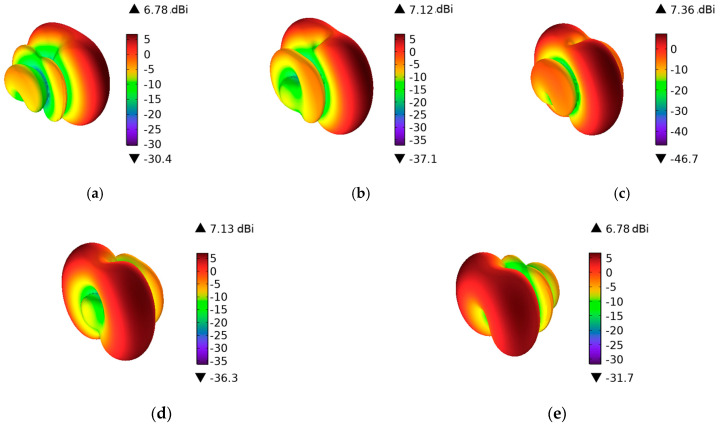
3D far-field radiation pattern while the unit phase variation is changing from −90 to 90 degrees in 45 degree steps. (**a**) Phase = −1.5708 rad; (**b**) Phase = −0.7854 rad; (**c**) Phase = 0 rad; (**d**) Phase = 0.7854 rad; (**e**) Phase = 1.5708 rad.

Figure 4 shows the three-dimensional far-field radiation pattern of the V2V antenna while the unit phase variation is changing from −90 to 90 degrees. The maximum gain is 7.36 dBi.

### 2.2. The Vehicle Model

**Figure 5:**
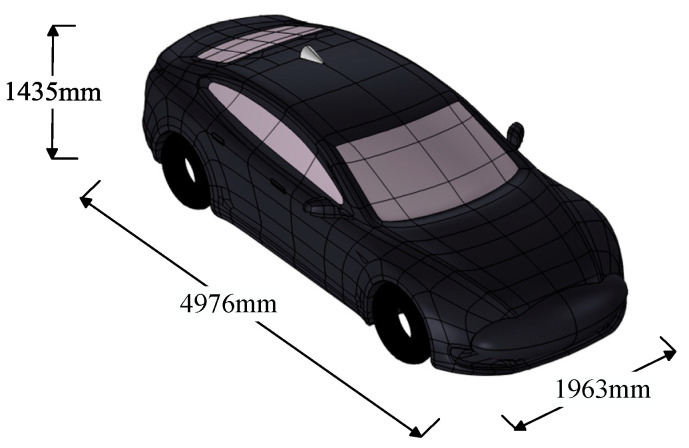
The dimensions of the car model.

A vehicle model was constructed using a commercially available electric vehicle as a reference. The model dimensions are 4976 mm × 1963 mm × 1435 mm, as shown in Figure 5. The model simplifies the car body and tires, with the car body primarily made of aluminum alloy and glass. The main material parameters are listed in Table 3.

The loss tangent (tanδ) is an important parameter for measuring the loss characteristics of materials. The complex dielectric constant of the material is calculated using Equation (2) [20]. The tires are composed of a mixture of rubber, carbon black, and other materials, and their actual loss characteristics are relatively complex. In this study, the loss tangent constitutive relation is used to model the tire domain as a lossy dielectric, accurately describing the material’s absorption and loss characteristics.

(2) $$\varepsilon_{r}=\varepsilon^{’}\left(1-j\tan\delta \right)$$

In Equation (2), $\varepsilon_{r}$ is the complex dielectric constant of the material, ε’ is the real part of the relative dielectric constant of the material, the relative permittivity of the tire is set to 2, and the loss tangent δ is set to 0.003 rad [21].

**Figure 6:**
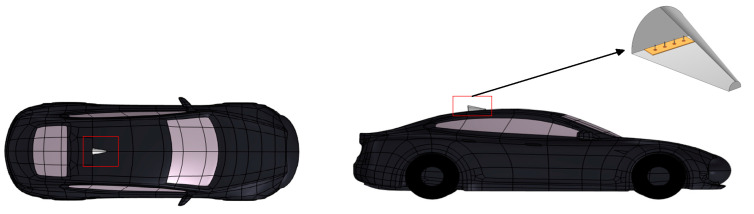
The relative position of the V2V antenna on the vehicle.

The V2V antenna is mounted at the rear of the car’s roof, inside a simple shark fin shell, as shown in Figure 6. The red square shows a shark fin antenna. The material of the shark fin shell is carbon fiber, of which the main material parameters are listed in Table 3.

### 2.3. The Human Model

**Figure 7:**
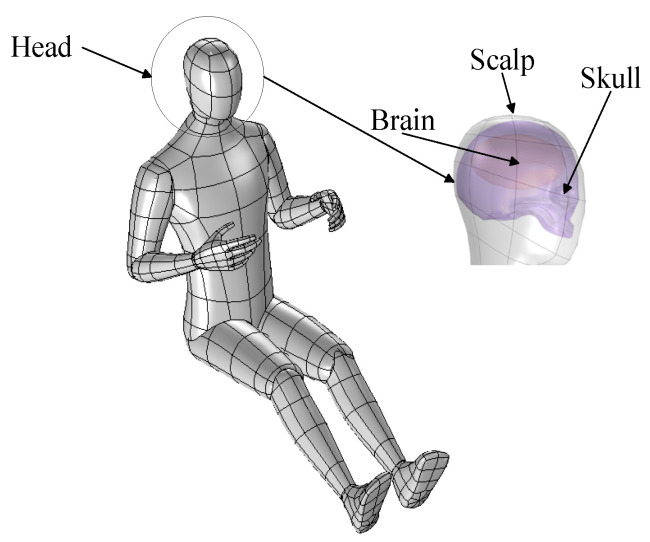
Human sitting posture model.

**Figure 8:**
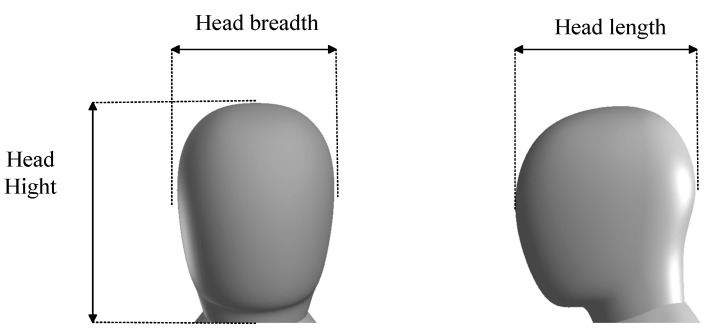
The dimensions of the head model.

In this paper, the primary objective is to analyze the electromagnetic exposure in the main regions of the human body. Simplifying secondary characteristics such as the ears can improve computational efficiency without significantly affecting the key results. Therefore, a simplified human body model was established in accordance with national standards [22]. The model has a height of 1.80 m, a seated height of 1.15 m, and a shoulder width of 0.419 m. This study employs a simplified head model, as shown in Figure 7. The model consists of the scalp, skull, and cerebrum, with the head length being 180 mm, head width 148 mm, and head height 217 mm, as shown in Figure 8 and Table 4. For the simplified human body model, the various tissues in the head model are simulated using the conductivity of similar tissues based on the frequency of the radiation source [23]. The relative permittivity and conductivity of the cerebrum are taken as the average values of gray matter, white matter, and cerebrospinal fluid, and the cerebrum is simplified as an ellipsoid. The relative permittivity and conductivity of the trunk are the average values of skin, bone, muscle, fat, and blood. The antenna, vehicle, and human body are all modeled in a spherical air domain. The air domain is truncated with a perfectly matched layer (PML) to absorb the radiated fields from the array structure.

## 3. Methods and Principles of Human RF Electromagnetic Exposure Assessment

### 3.1. Theoretical Basis of Electromagnetic Field

The study of computational electromagnetics is interdisciplinary and comprehensive, requiring large-scale computational speed and memory, as well as the application of different computational methods. The region to be computed for electromagnetic field problems typically consists of multiple media, so boundary conditions must be applied at the interfaces between the media. Boundary conditions describe the discontinuities in the electromagnetic field vectors at the interfaces and reflect the influence of the media boundaries on the propagation of electromagnetic waves. The definition of boundary conditions is shown in Equation (3).

(3) $${\left(\nabla \times E\right)}_{tan}=jk_{0}E_{tan}-\frac{j}{k_{0}}\left(\nabla_{tan}\times E_{tan}\right)+\frac{j}{k_{0}}\nabla_{tan}\left(\nabla_{tan}\cdot E_{tan}\right)$$

To perform the safety assessment of RF electromagnetic exposure in vehicles, the RF module in COMSOL was utilized to solve Maxwell’s equations with applied boundary conditions, obtaining the distribution of the induced electric field, as shown in Equation (4).

(4) $$\nabla \times \mu_{r}^{-1}\left(\nabla \times E\right)-k_{0}^{2}\left(\varepsilon_{r}-\frac{j\sigma }{\omega \varepsilon_{0}}\right)E=0$$

In the equation, $\mu_{r}$ is the relative permeability and *k*_0_ is the wave number in free space, where *k*_0_ $=\frac{\omega }{c}$.

### 3.2. Dielectric Parameters of Human Tissue

In 1941, Kenneth S. Cole and Robert H. Cole developed the mathematical theoretical model for the dielectric properties of biological tissues, known as the Cole–Cole model, which shows that conductivity or relative permittivity depends on frequency [24,25].

The dielectric properties (conductivity σ, relative permittivity ε) of human tissues are frequency-dependent. In 1996, S. Gabriel et al. derived the fourth-order Cole–Cole model based upon the research of R.H. Cole and K.S. Cole. In their three significant papers [26,27,28], they detailed the foundational theory of the dielectric properties of biological tissues, important experimental data, and the selection of fourth-order Cole–Cole model parameters when fitting human tissues.

For frequencies ranging from 10 Hz to 100 GHz, σ and ε can be calculated using the fourth-order Cole–Cole model [26,27,28], as shown in Equation (5).

(5) $${\hat{\varepsilon }}_{r}=\varepsilon_{r}^{’}-j\varepsilon_{r}^{\prime \prime }=\varepsilon_{r\infty }+\sum_{n=0}^{4}\frac{∆\varepsilon_{n}}{1+{\left(j\omega \tau_{n}\right)}^{1-\alpha_{n}}}+\frac{\sigma_{i}}{j\omega \varepsilon_{0}}$$

In the equation, ω=2πf represents the angular frequency, with units of rad/s; $\hat{\varepsilon }$_r_ is the complex relative permittivity, with units of F/m, indicating the dielectric response of a material to an electric field per unit length; $\varepsilon_{r}’$ is the real part of the complex relative permittivity, and $\varepsilon_{r}\prime \prime$ is the imaginary part of the complex relative permittivity; $\varepsilon_{r\infty }$ is the relative permittivity at optical frequencies; $\sigma_{i}$ is the static conductivity, with units of Siemens per meter (S/m); $\tau_{n}$ is the relaxation time constant for the n-th dispersion region; ${∆\varepsilon }_{n}$ is the increment of the dielectric constant in the n-th dispersion region relative to the static dielectric constant; and $\varepsilon_{0}$ = 8.854187817 × 10^12^ is the permittivity of free space (F/m).

The fourth-order Cole–Cole model is adopted to calculate the dielectric properties at 5.9 GHz, a frequency critical for V2V communications. In this paper, the dielectric parameters of the trunk are taken as the unweighted arithmetic average of the dielectric parameters of five tissues: skin, bone, muscle, fat, and blood. For the cerebrum, the unweighted arithmetic average dielectric parameters of gray matter, white matter, and cerebrospinal fluid are used, and the calculated dielectric parameters of head and human trunk models are shown in Table 5.

RF antennas in free space utilize ambient electromagnetic radiation. When a mobile device operates at a specific frequency, it induces an electromagnetic field around the human body due to the influence of the external electromagnetic field. The characteristics of the body tissues consume the induced electromagnetic field, absorbing and dissipating the electromagnetic energy, which leads to an increase in the internal temperature of the tissues. The internationally recognized specific absorption rate (SAR) is used to measure the amount of electromagnetic radiation energy absorbed or consumed by human body tissues [29,30]. The mathematical expression of SAR is shown in Equation (6).

(6) $$SAR=\frac{d}{dt}\left(\frac{dW}{dm}\right)=\frac{d}{dt}\left(\frac{dW}{\rho dV}\right)$$

where *t* is the radiation time (s); W is the radiated power (w); m is the tissue mass (kg); *ρ* is the tissue density (kg/m^3^); and V is the tissue volume (m^3^). Another expression of SAR is Equation (7), where σ is the tissue electrical conductivity (S/m).

(7) $$SAR=\frac{\sigma }{2\rho }{\left|E\right|}^{2}$$

The human head tissues absorb electromagnetic radiation under RF exposure and convert EM radiation into Joule heat.

This study employs Pennes’ transient bioheat equation within the COMSOL platform to simulate dynamic heat transfer characteristics during electromagnetic wave penetration from the scalp layer through to the skull layer and cerebrum layer [31,32], as follows:

(8) $$\rho C\frac{\partial T}{\partial t}=\nabla \cdot \left(k\nabla T\right)+\rho_{b}C_{b}\omega_{b}\left(T_{b}-T\right)+Q_{met}+Q_{ext}$$

where *ρ* is the tissue density (kg/m^3^); *C* is the specific heat capacity of the tissue (J/(kg·°C)); *k* is the thermal conductivity (W/(m·°C)); *T* is the tissue temperature (°C); *T_b_* is the blood temperature (°C), which is set to 37 °C; $\rho_{b}$ = 1049.75 is the blood density (kg/m^3^); *C_b_* = 0.52 is the specific heat capacity of the blood (J/(kg·°C)); $\omega_{b}$ is the blood perfusion rate (s⁻^1^); *Q_met_* is the metabolic heat source (W/m^3^); and *Q_ext_* is the external heat source (W/m^3^).

The heat transfer between tissue and blood flow is approximated as $\rho_{b}$*C_b_*$\omega_{b}$(*T_b_* − *T*). The external heat source represents the heat loss due to biological tissue absorbing electromagnetic radiation, as shown in Equation (9).

(9) $$Q_{ext}=\frac{1}{2}\sigma_{tissue}{\left|E\right|}^{2}=\rho \cdot SAR$$

where $\sigma_{tissue}=2\pi$*f*$\varepsilon_{r}’\varepsilon_{0}$; $\varepsilon_{r}$ is the relative dielectric constant of the tissue; and $\varepsilon_{0}$ is the (absolute) dielectric constant in a vacuum.

In this study, the thermal parameters of various tissue layers used in analyzing the temperature rise in the human head model are shown in Table 6 [33].

### 3.3. Simulation Calculation Methods

This study uses COMSOL Multiphysics simulation software based on the finite element method (FEM) for multi-physics coupling. The finite element method primarily divides the problem domain into a mesh of many connected small subdomains, referred to as finite element domains. By solving the values at the boundary points of each mesh, the equilibrium conditions for the entire domain are derived, and the solution for the entire domain is obtained. The basic principles of FEM are the variational principle and the weighted residual method. In FEM computations, the key step is to introduce interpolation functions, which represent the solution of each finite element mesh, allowing the solution for the entire domain to be determined. However, this process does not require introducing boundary conditions for each finite element mesh after each computation, but instead, boundary conditions are applied after solving the entire domain.

**Figure 9:**
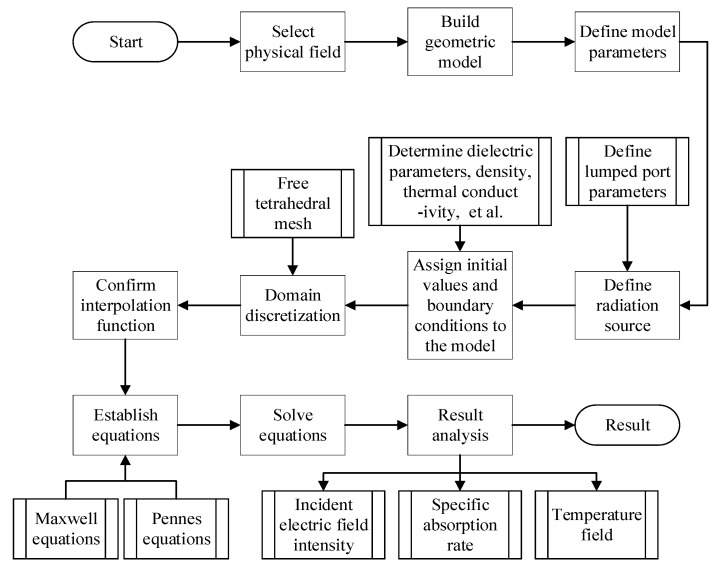
COMSOL solution process.

This study employs frequency-domain and transient coupling analysis for multi-physics simulations, using COMSOL Multiphysics. The frequency-domain study is set with a center frequency of 5.9 GHz, while the temperature rise process over a 30-min period is simulated through transient analysis. The solution process is shown in Figure 9.

**Figure 10:**
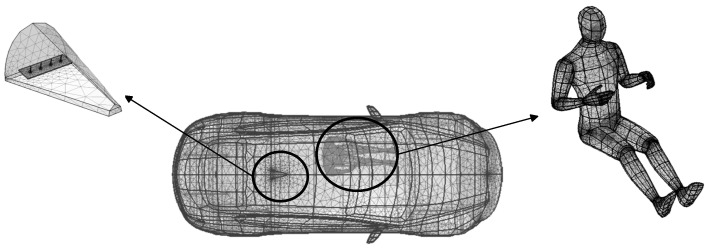
Mesh discretization of the 3D geometric model.

Mesh generation of finite element model [34,35]: After constructing the vehicle model, human body model, and RF V2V antenna in COMSOL, a 3D geometric model needs to be meshed. In this study, a free tetrahedral mesh is used for discretization. Considering both accuracy and the computational capacity of the computer, finer mesh divisions are applied to smaller structures, more complex human body models, and antenna models, as shown in Figure 10.

### 3.4. ICNIRP Guidelines

To assess the impact of electromagnetic environments on the human body, the International Commission for Non-Ionizing Radiation Protection (ICNIRP) has provided reference levels for exposure to electromagnetic fields in the frequency range of 100 kHz to 300 GHz. This paper primarily refers to the exposure limits in the ICNIRP guidelines. The ICNIRP standard, established in 1998, set basic exposure limits for different frequency ranges based on various scientific evidence, and was later revised in 2010 and 2020. In the ICNIRP guidelines, electromagnetic exposure limits are distinguished into reference levels, which are related to the measurement environment, and basic restrictions applying to the biological effects within the body [17,36,37]. The ICNIRP standards provide a reference for limiting electromagnetic field exposure and preventing adverse health effects from electromagnetic environments, as shown in Table 7.

According to the ICNIRP guidelines, whole-body average SAR is to be averaged over 30 min. Local SAR is to be averaged over a 10-g cubic mass. This paper focuses on electromagnetic exposure safety assessment for drivers, and therefore only the occupational exposure limits outlined in the ICNIRP guidelines are taken into consideration. Moreover, in the case of occupational exposure, the increase in the core body temperature must not exceed 1 °C.

## 4. Safety Assessment of In-Vehicle Human EMF Exposure Under V2V Communications

### 4.1. Electric Field Intensity Distribution of Vehicles

**Figure 11:**
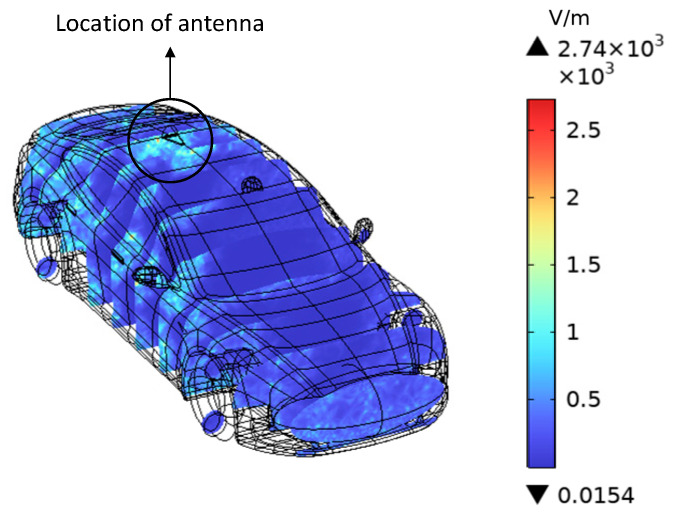
Surface distribution of electric field intensity of the vehicle.

The distribution and magnitude of the induced electric field intensity on the vehicle body surface remain similar when the phase of the excitation current of the V2V antenna is varied. This paper only presents the induced electric field distribution when the phase of the antenna excitation current is 0 radians. Figure 11 shows distribution of the induced electric field intensity on the surface of the electric vehicle, with the maximum induced electric field intensity occurring in the area covered by the shark fin antenna shell on the vehicle’s roof.

### 4.2. Human Electric Field Intensity Distribution

**Figure 12:**
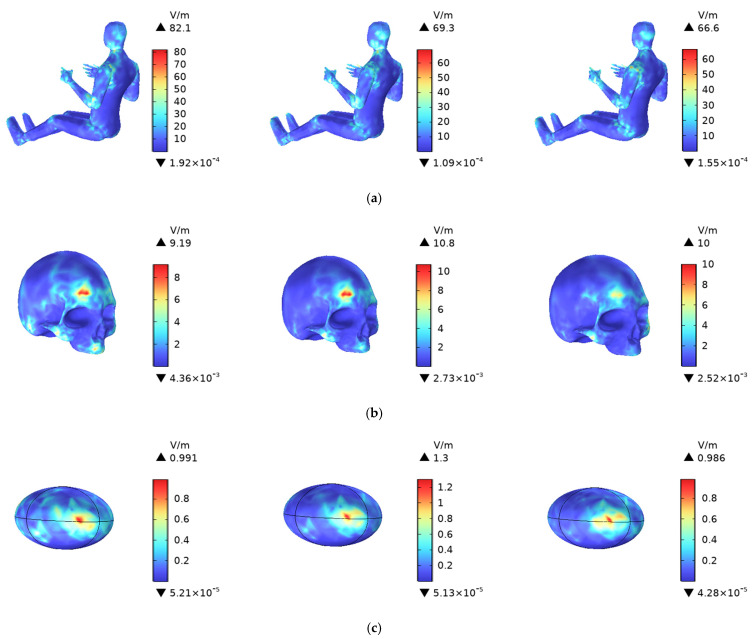
Distribution of induced electric field strength in the trunk and head of the human body, where the phase of the radiation source element changes from −90 degrees to 0 degrees, with a step size of 45 degrees. (**a**) for the Trunk, (**b**) for the Skull, and (**c**) for the Cerebrum.

In this paper, the antenna model is a phased array antenna. Figure 12 illustrates the distribution of induced E-field intensity in the trunk and head of the human body model when the V2V antenna operates at the maximum allowable radiation power of 33 dBm, under different phase settings.

As shown in Figure 12, the induced E-field intensity in the trunk is relatively dispersed and not concentrated. The upper body is in closer proximity to the radiation source, and the antenna’s radiation pattern is oriented more directly toward the upper body of the human driver, leading to stronger electromagnetic field intensity and higher energy absorption. The maximum value of 82.1 V/m occurs when the excitation phase of the radiation source is −90 degrees, with higher values predominantly located in the left shoulder.

The induced electric field strength in the skull shows little variation with changes, with higher values concentrated in the right frontal and occipital regions. The induced electric field intensity distribution in the cerebrum is irregular, with higher values mainly concentrated in areas closer to the antenna.

### 4.3. Human SAR Distribution

**Figure 13:**
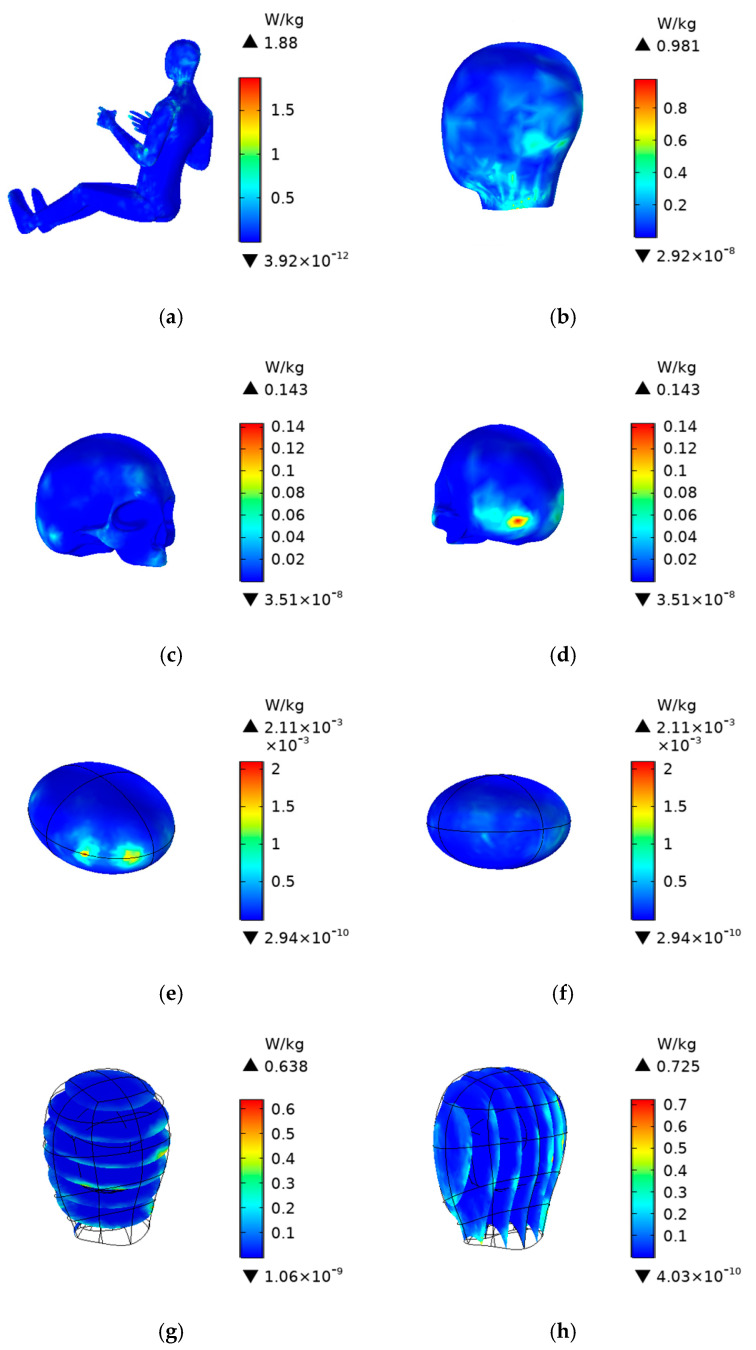
Specific absorption rate (SAR) of the human body (**a**) for the whole body, (**b**) for the scalp, (**c**,**d**) for the skull, (**e**,**f**) for the cerebrum, (**g**) for the XY section of head, and (**h**) for the YZ section of head.

For RF radiation, the externally applied electric field induces an electric field within the human body. Since human tissues have resistive properties, the electric field generates currents, thereby absorbing and dissipating electromagnetic energy, ultimately leading to thermal effects. According to ICNIRP guidelines, the 30-min SAR limit for professionals, such as drivers, who are exposed to electromagnetic fields in the workplace for extended periods is 0.4 W/kg. The maximum SAR limit for localized exposure in the head and torso is 10 W/kg, while for the limbs, it is 20 W/kg. These thresholds have been widely applied in recent studies on biological electromagnetic exposure. Since the SAR values change insignificantly with variations in the radiation source phase, and because the radiation direction is more aligned with the driver’s position when the excitation current phase is −1.5708 rad, this paper only presents the relevant SAR distribution and average values when phase is −1.5708 rad. The SAR distribution for the whole body, head, skull, and cerebrum of the driver after 30 min of exposure is shown in Figure 13.

As shown in Figure 13, the whole-body SAR distribution is relatively dispersed, with higher values primarily concentrated in the upper body. The maximum value is 1.88 W/kg. The higher values of the SAR_10g_ for the scalp are mainly located in areas closer to the V2V antenna, with a maximum value of 0.981 W/kg. For the skull, the higher values of SAR_10g_ are concentrated in the occipital region, with a maximum value of 0.143 W/kg. The maximum SAR_10g_ value in the cerebrum is 0.00211 W/kg, which is also well below the maximum limit specified by the ICNIRP limits.

### 4.4. The Temperature Field Distribution of the Human Head

**Figure 14:**
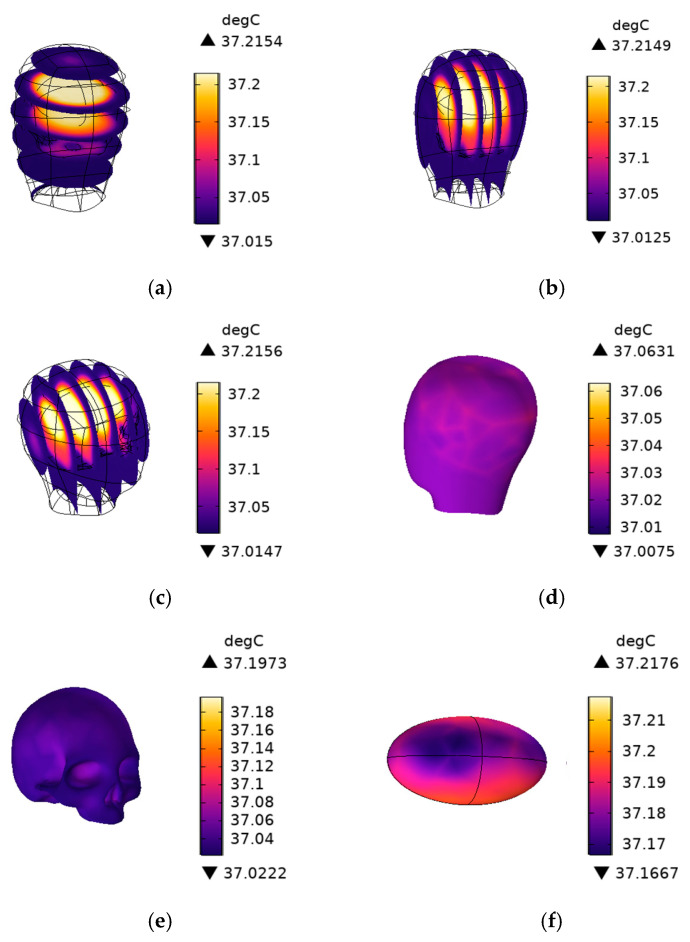
Temperature distribution section of the head model with 30 min irradiation. (**a**) for the XY section of head, (**b**) for the YZ section of head, (**c**) for the ZX section of head, (**d**) for the scalp, (**e**) for the skull, and (**f**) for the cerebrum.

The temperature distribution cross-section of the head model after 30 min of exposure is shown in Figure 14. When the electromagnetic waves generated by the onboard V2V antenna penetrate the human body, various tissue layers absorb electromagnetic radiation energy due to electromagnetic coupling effects and the cumulative effects of radiation over time. This energy is subsequently converted into heat, potentially causing an increase in tissue temperature. The initial temperature is set to 37 °C. The maximum temperature rise occurs in the cerebrum due to its higher metabolic heat source *Q_met_* (7100 W/m^3^). Although the specific absorption rate of the brain is lower than that of the skull and scalp, its higher metabolic heat source leads to the accumulation of heat locally, thereby resulting in a more significant temperature rise.

The maximum temperature rise is 0.2176 °C, occurring locally in the cerebrum, which is below the temperature rise limit of 1 °C specified in the ICNIRP guidelines (2020).

**Figure 15:**
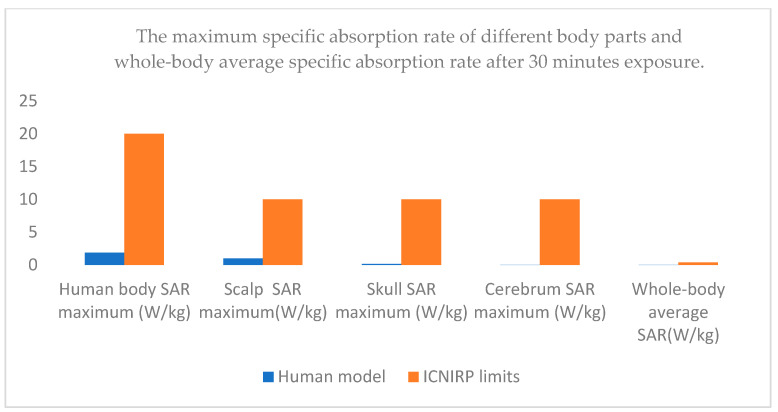
Comparison of the maximum SAR values for different parts of the human body and the whole-body average SAR after 30 min of exposure with the ICNIRP limits.

Table 8 presents a comparison of the whole-body average SAR, the maximum SAR values in various tissues, the maximum head temperature rise, and the ICNIRP limits after 30 min of exposure, which is shown intuitively and clearly in Figure 14 and Figure 15. The results of this study show that the SAR10g values are of the same order of magnitude as those reported in [12] (i.e., on the order of hundreds of mW/kg). A key difference, however, is that this study also investigates the temperature field distribution, which was not considered in [12].

**Figure 16:**
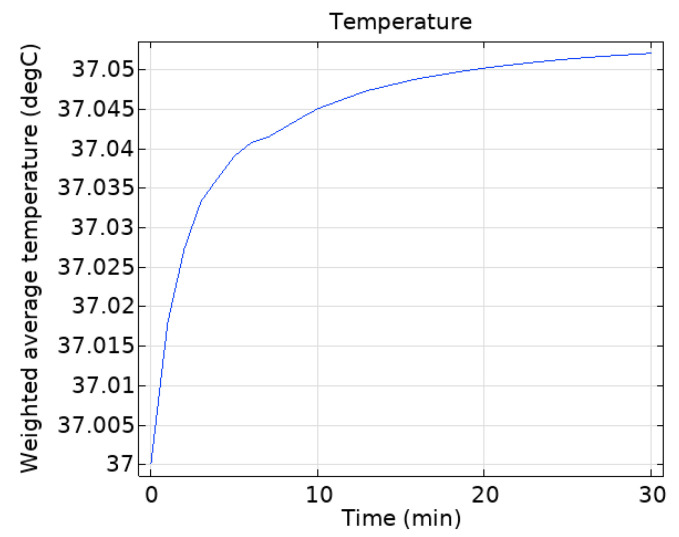
Variation of average human head temperature over time under 30 min of electromagnetic radiation.

Figure 16 shows the weighted average temperature rise in the head after 30 min of exposure. The 30 min average human core temperature rise is 0.055 °C.

## 5. Conclusions

This paper proposes a monopole array antenna for V2V communication and evaluates electromagnetic exposure at the maximum permitted radiated power (33 dBm) using an adult male model. The exposure levels in the trunk, scalp, skull, and brain under this electromagnetic environment are quantified and calculated. Using the frequency-domain and transient coupling techniques in COMSOL Multiphysics, and employing multi-physics simulations, the study focuses on the distribution of SAR and temperature fields in the human body.

The main objective of this study is to systematically analyze the potential hazards caused by EM fields generated by the RF V2V antennas. The peak value of the induced electric field in the human body is 82.1 V/m. The average whole-body specific absorption rate over 30 min is 0.008728 W/kg, which is 2.18% of the ICNIRP limit. The maximum local average specific absorption rate for 10 g (SAR_10g_) of tissue in the head is 0.981 W/kg, which is below the limit for occupational, 9.81% of the ICNIRP limit. The maximum human core temperature increase after 30 min is 0.2176 °C, which is 21.76% of the ICNIRP temperature rise limit.

Numerical simulations under specific conditions demonstrate that the radiation levels of V2V antennas comply with ICNIRP safety limits, which is considered safe for human health. However, cumulative exposure effects become significant with prolonged operation. Therefore, when designing and utilizing V2V antennas and systems, it is crucial to take various factors into account for regulation and restriction to guarantee adherence to human exposure safety thresholds.

However, the research has some limitations regarding the models, scenarios, and other factors. The simplified human body model was employed primarily to evaluate electromagnetic exposure in the head and torso regions. This computational model excludes high-resolution modeling of sensitive organs. Moreover, the study assumes stationary vehicles, differing from real-world dynamic driving conditions.

Future research will focus on the following aspects:

(1) Design of MIMO and Millimeter-Wave Antennas for V2V communications;

(2) Antenna-vehicle chassis integration effects through full-wave EM simulations and measurements;

(3) Realistic human-antenna modeling for V2V radiation safety assessment;

(4) Passenger EM exposure analysis with seating-position-dependent dose evaluations.

Ongoing research on the radiation effects of V2V antennas will deepen our understanding of potential risks and help ensure electromagnetic exposure levels consistently remain within established safety limits, thereby safeguarding human health.

## Figures and Tables

**Table 1:** Material parameters of the antenna.

Name	Relative Permittivity	Relative Permeability	Electrical Conductivity (S/m)
Quarter-wave monopole antenna	1	1	$5.998\times 10^{7}$
Coaxial lumped port	3.38	1	0
Dielectric substrate	3.38	1	0

**Table 2:** Excited phase variation on each antenna element in degree.

Unit Phase (α)	Lumped Port 1	Lumped Port 2	Lumped Port 3	Lumped Port 4
−90	0	−90	−180	−270
−45	0	−45	−90	−135
0	0	0	0	0
45	0	45	90	135
90	0	90	180	270

**Table 3:** Parameters of vehicle materials.

Name	Relative Permittivity	Relative Permeability	Electrical Conductivity (S/m)	ρ (kg/m^3^)	k (W/(m·°C))	C (J/(kg·°C))
aluminum alloy	1	1	$4.032\times 10^{6}$	2730	44.5	475
glass	4.2	1	$10^{-14}$	2210	1.4	730
carbon fiber	2	1	5000	100	5	1.2

**Table 4:** The dimensions of the head model.

Name	Human Model (mm)
Head height	217
Head length	180
Head breadth	148

**Table 5:** Dielectric parameters of different human tissues.

Human Tissues	Relative Permittivity	Electrical Conductivity (S/m)
Scalp	35.03	3.80
Skull	9.97	1.22
Brain Grey Matter	43.86	5.10
Brain White Matter	32.52	3.58
Cerebrospinal Fluid	60.28	8.00
Cerebrum	45.55	5.56
Skin	35.03	3.80
Bone	15.32	2.19
Muscle	48.35	5.08
Fat	4.95	0.30
Blood	52.36	6.65
Trunk	31.20	3.60

**Table 6:** Thermal parameters of human head tissues.

Human Tissues	ρ (kg/m^3^)	*k* (W/(m·°C))	*C* (J/(kg·°C))	$Q_{met}$ (W/m^3^)	$\omega_{b}$ (s^−1^)
Scalp	1125	0.42	3600	1620	0.02
Skull	1990	0.37	3100	610	0.00046
Cerebrum	1038	0.53	3650	7100	0.00883

**Table 7:** Basic restrictions for electromagnetic field exposure from 100 kHz to 6 GHz, for averaging intervals ≥6 min.

Exposure Scenario	Frequency Range	Whole-Body Average SAR (W·kg^−1^)	Local Head/Torso SAR (W·kg^−1^)	Local Limb SAR (W·kg^−1^)
Occupational	100 kHz–6 GHz	0.4	10	20
General public	100 kHz–6 GHz	0.08	2	4

**Table 8:** Maximum SAR values, whole-body average SAR, and maximum temperature rise for different parts of the human body after 30 min of exposure, compared with ICNIRP limits.

	Human Model	ICNIRP Limits
Human body SAR maximum (W/kg)	1.88	20
Scalp SAR maximum (W/kg)	0.981	10
Skull SAR maximum (W/kg)	0.143	10
Cerebrum SAR maximum (W/kg)	0.00211	10
Whole-body average SAR (W/kg)	0.008728	0.4
Head temperature rise maximum (°C)	0.2176	1

## Data Availability

The data in this study are available from the corresponding author upon request.
